# Anodal transcranial patterned stimulation of the motor cortex during gait can induce activity-dependent corticospinal plasticity to alter human gait

**DOI:** 10.1371/journal.pone.0208691

**Published:** 2018-12-21

**Authors:** Satoko Koganemaru, Yusuke Mikami, Hitoshi Maezawa, Masao Matsuhashi, Satoshi Ikeda, Katsunori Ikoma, Tatsuya Mima

**Affiliations:** 1 Department of Rehabilitation Medicine, Hokkaido University Hospital, Sapporo, Japan; 2 Human Brain Research Center, Graduate school of Medicine, Kyoto University, Kyoto, Japan; 3 Department of Oral Physiology, Division of Oral Functional Science, Graduate School of Dental Medicine, Hokkaido University, Sapporo, Japan; 4 The Graduate School of Core Ethics and Frontier Sciences, Ritsumeikan University, Kyoto, Japan; University of Ottawa, CANADA

## Abstract

The corticospinal system and local spinal circuits control human bipedal locomotion. The primary motor cortex is phase-dependently activated during gait; this cortical input is critical for foot flexor activity during the swing phase. We investigated whether gait-combined rhythmic brain stimulation can induce neuroplasticity in the foot area of the motor cortex and alter gait parameters. Twenty-one healthy subjects participated in the single-blinded, cross-over study. Each subject received anodal transcranial patterned direct current stimulation over the foot area of the right motor cortex during gait, sham stimulation during gait, and anodal transcranial patterned direct current stimulation during rest in a random order. Six subjects were excluded due to a failure in the experimental recording procedure. Complete-case analysis was performed using the data from the remaining 15 subjects. Self-paced gait speed and left leg stride length were significantly increased after the stimulation during gait, but not after the sham stimulation during gait or the stimulation during rest. In addition, a significant increase was found in the excitability of the corticospinal pathway of the left tibialis anterior muscle 30 min after stimulation during gait. Anodal transcranial patterned direct current stimulation during gait entrained the gait cycle to enhance motor cortical activity in some subjects. These findings suggest that the stimulation during gait induced neuroplasticity in corticospinal pathways driving flexor muscles during gait.

## Introduction

During human bipedal locomotion, cortical and supraspinal projections control posture and leg movements in coordination with the spinal locomotor system [1]. Compared with the quadrupedal gait of animals, the biomechanical need for an erect bipedal gait appears to have further encephalized the neural control of human walking [1, 2].

Phasic corticospinal excitability has been found to modulate motoneuronal circuits during walking [1, 3, 4]. The corticospinal neurons show increase of activity in controlling the flexor muscles during walking, but this increase is not often seen in controlling the extensor muscles [4, 5]. A significant coherence between the primary motor cortex (M1) and flexor muscles, such as the tibialis anterior (TA), is found prior to heel strike during the swing phase of walking [6]. Stroke patients with lesions in the sensorimotor cortex or corticospinal tracts often exhibit drop-foot during the swing phase [7]. These findings suggest that corticospinal inputs contribute to the phasic control of swing-related activities in foot flexor muscles.

Patterned rhythmic transcranial direct current stimulation (tDCS) such as transcranial alternating current stimulation (tACS) [8–12] and tACS with a constant direct current (DC) offset [13–15], are noninvasive methods of modulating brain activity. These approaches can modulate intrinsic brain rhythmicity, thereby altering sensorimotor and cognitive functions [16–22]. Applying tACS over the M1 at 20 Hz slows voluntary arm movements by entraining cortical beta-band activity [20]. Therefore, offering tACS at a similar frequency to that of resting movement could control tremor in patients with Parkinson’s disease [23]. The use of tACS with a constant positive DC offset over the M1 has been shown to increase corticospinal excitability, with the effect being sustained for more than 20 min [13].

Human bipedal gait is a regular movement based on brain and spinal rhythmicity [1, 4, 24]. However, no reports published thus far have indicated whether patterned tDCS can modulate brain rhythmicity during gait to enhance corticospinal activity-associated gait control. If patterned tDCS can alter gait-associated corticospinal activity, the method may become a promising therapeutic approach in the recovery of gait function for patients with central nervous system disorders. In previous studies, patterned tDCS was found to entrain and enhance brain activity; however, the method was not used in a time-locked way and the frequency parameters of the tDCS were unrelated to intrinsic brain rhythmicity [14, 15, 17–22]. In this study, we hypothesized that the use of patterned tDCS over the M1 foot area at a frequency similar to a normal gait frequency, applied in a non-time-locked way and without relation to individual gait cycles, may induce plasticity of the M1 and modulate gait parameters. We assumed that the frequency of patterned tDCS similar to a normal gait frequency might be also similar to that of the cortical activity associated with stepping during gait. The main outcome measure was gait parameters, and the secondary measure was M1 excitability controlling a tibialis anterior (TA) muscle as one of foot flexor muscles.

## Methods

### Experimental protocol

#### Participants

Twenty-three healthy volunteers were recruited in the study using the Web advertisement of Kyoto University (URL: http://www.s-coop.net/service/arbeit/). Inclusion criteria was no history of chronic or acute neurological, psychiatric, or medical diseases; no family history of epilepsy; no present pregnancy; no cardiac pacemaker; no previous surgery involving implants (aneurysm clips and brain or spinal electrodes), and absence of acute or chronic medication or drug intake. Based on the consideration of inclusion criteria, two volunteers were excluded and only 21 healthy individuals (nine women and 12 men) aged 20–40 years [mean ± standard deviation (SD), 25.9 ± 7.0 years] were included in the study. The sample size was determined for detecting the effect of M1 oscillatory tDCS according to the previous reports [8, 13, 20, 25]. All were right-handed according to the Edinburgh handedness inventory [26] and had right foot preference according to the Chapman test [27]. The study protocol was approved by the Committee of Medical Ethics of the Graduate School of Medicine, Kyoto University, Japan (C-800) and written informed consent was obtained from all subjects.

#### Electromyogram (EMG) recording

EMGs were recorded from the right and left TA muscles (foot flexors) and gastrocnemius (GC) muscles (foot extensors) using pairs of silver electrodes in a belly-tendon montage. A pair of electrodes for GC muscles was pasted on the surface of lateral head of gastrocnemius muscle. The EMG was amplified, filtered (bandpass, 5–1,000 Hz), and digitized at a sampling rate of 10 kHz using the Map1496 system (Nihon-Santeku Co., Osaka, Japan).

#### Transcranial magnetic stimulation (TMS) procedures

For TMS, each subject was seated comfortably in an armchair. Focal TMS was performed using a double-cone magnetic coil (outer diameter of each wing, 14 cm) connected to a Magstim 200 magnetic stimulator (Magstim, Whitland, Dyfed, UK). The center of the junction region of the coil was placed tangentially to the scalp and 90° lateral to the midline, and stabilized during the measurement. The coil was held so that currents in the brain flowed rightward for stimulation of the left M1 and leftward for stimulation of the right M1 [28].

The optimal TMS coil scalp positions for induction of motor responses from bilaterial TA and GC muscles were determined for each subject. The resting motor threshold (rMT) was defined as the minimum stimulator output eliciting motor evoked potentials (MEPs) > 50 μV in five out of 10 consecutive pulses [29]. The rMTs were represented as percentage of the maximum stimulator output.

To assess corticospinal excitability, MEP amplitudes were measured with TMS intensity fixed to produce a MEP of approximately 1 mV from bilateral TA and GC muscles (stimulus intensity, SI1 mV) at rest using the Magstim 200 apparatus. TMS intensities with SI1 mV as percentage of the maximum stimulator output were recorded for bilateral TA and GC muscles in the baseline. Complete muscle relaxation was continuously monitored by visual feedback of surface EMGs. The peak-to-peak amplitudes of the MEPs were measured in each single trial and averaged for each subject.

To investigate the motor inhibitory system, the cortical silent period (CSP) was assessed for the bilateral TA and GC muscles. CSP is considered to reflect inhibitory GABA_B_-ergic neuron activities in the M1 area [30]. TMS pulses were given with a stimulation intensity of 140% rMT during isometric submaximal contraction. After the maximum EMG amplitudes were measured during the maximum contraction, subjects were asked to maintain the contraction so that around 40% of the maximum EMG amplitudes were kept. During the contraction, visual check of the raw EMG data was performed by the experimenter. If the EMG amplitudes did not reach the targeted level, subjects were asked to strengthen their muscle contraction. The EMG amplitudes were not always kept at exactly 40% of the maximum EMG amplitudes since the level of background activation has no effect on the CSP duration [31, 32]. The duration of the CSP was defined as the time from TMS onset until return of voluntary EMG activity and it was manually analyzed by the experimenters [30]. Each muscle was measured in a random order.

The rMT was 51.9% ± 10.9% of the maximum stimulator output for the left TA muscle, 52.3% ± 10.6% for the left GC, 53.0% ± 10.0% for the right TA muscle, and 53.1% ± 10.0% for the right GC muscle. The stimulator intensities required to elicit an MEP of 1 mV (SI1 mV) were 69.4% ± 14.8% for the left TA muscle, 76.1% ± 14.6% for the left GC, 74.5% ± 12.4% for the right TA muscle, and 77.7% ± 12.6% for the right GC muscle.

#### Gait analysis

To investigate the effects on basic walking function, a 10-meter walk test was performed. Subjects were asked to walk at their preferred walking speed along a 14-meter walkway without any break to the end point while wearing their shoes. To eliminate acceleration and deceleration periods, subjects started and ended their laps 2 meter before and beyond the middle 10 meter of walkway. Subjects were videotaped at a rate of 30 frames per second in the sagittal plane as they walked (GZ-E355, JVC KENWOOD Co., Ltd., Japan). The time to cover the 10 m of the walkway was measured by frame-by-frame advance function and the mean distance of 8 strides of the left leg (i.e., from one left foot floor contact to the next) was measured by marking the start and end point in a freeze-frame in offline (Everio MediaBrowser 4, JVC KENWOOD Co., Ltd., Japan; Adobe Photoshop CS6, Adobe Systems Co., Ltd., Japan.). The participants were asked to perform two trials in the 10-meter walk test. The individual self-paced 10-meter walk speed and the left stride length were calculated from the two trials.

The evaluation was performed before, immediately after, and 30 min after the intervention (designated as the “pre,” “post0,” and “post1” time points, respectively), which required approximately 15 min.

### Interventional protocol

**Fig 1:**
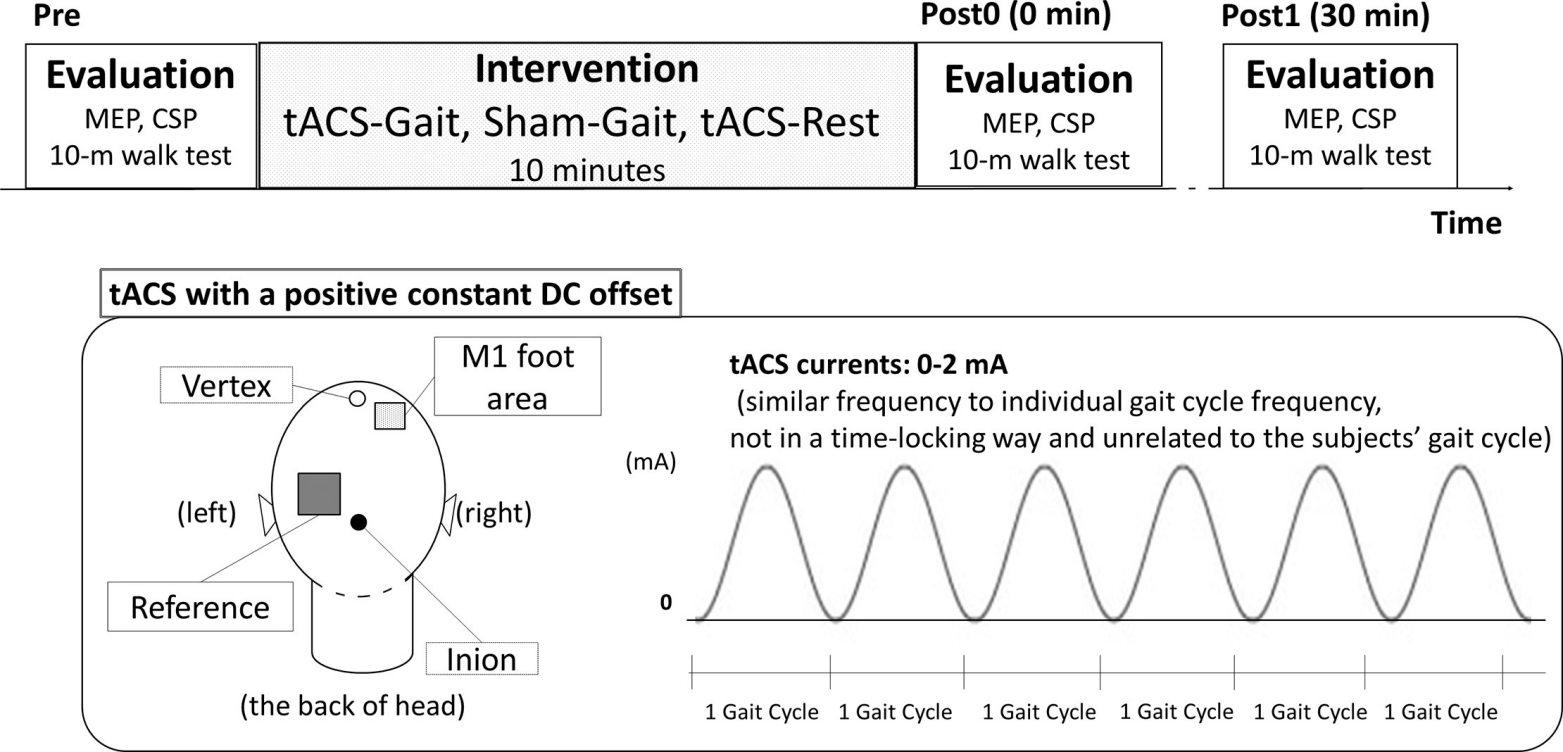
Interventional protocol. Three experimental interventions were performed: (1) tACS of the right M1 foot area during treadmill gait (tACS-Gait) (2) sham tACS during treadmill gait (Sham-Gait) and (3) tACS of the left M1 foot area during rest (tACS-Rest). Corticospinal excitability was evaluated by transcranial magnetic stimulation (TMS)-induced motor evoked potentials (MEPs) before, immediately after, and 30 min after the intervention (designated as the ‘pre’, ‘post0’, and ‘post1’ time points, respectively). Gait parameters were evaluated before and 30 min after the intervention.

Subjects first performed a 4-min walk on a treadmill (DK-208, Daikou Co., Ltd., Japan) to confirm individual preferred gait frequency. Then subjects performed a 10-min walk at this comfortable pace during the tACS-Gait intervention. The electrical currents for tACS with a constant DC offset were delivered through a neuroConn DC Stimulator (Ilmenau, Germany). The stimulation current waveform was a sinusoidal wave of 2 mA (from 0 mA to +2 mA) peak-to-peak amplitude delivered at the approximate gait frequency (within ±0.01 Hz). As for stimulation parameters, we chose the sinusoid waveform of tACS currents because it was safely used and effectively modified brain activity in previous studies [9, 25, 26]. tACS currents were not given in a time-locking way and was unrelated to the subjects’ gait cycle during the intervention. tACS with a constant DC offset lasted for the entire 10-min walk plus fade-in and fade-out periods of 10 cycles. For tACS with a constant DC offset over the right M1 foot area, the electrode (3 × 3 cm) was centered on the scalp position where the TMS coil elicited the largest and the most reliable motor response in the left TA muscle. The reference electrode (5 × 5 cm) was centered 3 cm left-lateral and 3 cm rostral from the inion over the occipital area (Fig 1).

In the sham stimulation condition, current was delivered only 10 cycles with electrodes positioned on the right M1 foot area with fade-in and fade-out periods of 10 cycles.

We conducted three interventional experiments: (1) tACS with a constant positive DC offset to the right M1 foot area during treadmill gait (tACS-Gait) as described, (2) sham tACS during treadmill gait (Sham-Gait), and (3) tACS with a constant positive DC offset on the right M1 foot area during rest (tACS-Rest). They were conducted in a random order on three different days, with a break of at least 5 days between each.

### Assessment of entrainment effects of tACS with a constant DC offset on gait parameters

We investigated the entrainment effects of tACS with a constant DC offset on gait cycle in the tACS-Gait condition. Individual gait cycle was assessed by an accelerometer placed on the middle of the lower back (BrainAmp system, Brain Products GmbH, Germany) so that the positive peak of acceleration indicated the initiation of the left leg stance phase. The tACS with a constant DC offset current waveform was recorded concurrently with the accelerometer to determine the phase difference by using the signal-out function of a DC Stimulator (Ilmenau, Germany).

### Assessment of rhythmic sensation during tACS with a constant DC offset

To confirm whether subjects felt any rhythmicity of the tACS currents, they were asked to push the button whenever they felt any sensation during tACS applied over the M1 area in the resting state on days different from those of the intervention. We simultaneously recorded the tACS currents and the timing of button-pushing for 5 min. This assessment was conducted one day between the 1^st^ and 2^nd^ session or between the 2^nd^ and 3^rd^ session in a random assignment to each subject with a break of at least 5 days before and after each session.

### Data analysis

To investigate a relationship between the changes in corticospinal excitability of the stimulated area and walking function in tACS-Gait, correlation coefficients were calculated between the change ratios (= post0/pre and post1/pre) of the MEP amplitudes from the left TA muscles, the gait speed and the left leg strides.

To determine whether pre-EMG activity was altered for CSP measurements before and after the interventions, the integral values (μV·sec) of the root mean square (RMS) of EMG activity (μV) for 100 msec before the TMS pulse were calculated.

For evaluation of the entrainment effect, recorded tACS currents were zero-phase bandpass filtered by using first order Butterworth filter in both directions in off-line at cut-off frequencies of 0.5 and 2 Hz. Analytic signals were acquired by the bandpass filtered currents following Hilbert transform.

The instantaneous phase of the initiation of the gait cycle in the left leg (the positive peak recorded by the accelerometer) was calculated during 10 min of treadmill gait. All instantaneous phases of the positive peak were represented by complex numbers and averaged. The absolute value and phase of the average were regarded as the phase synchronization index (PSI) and mean phase difference (MPD), respectively. A PSI of 0 represents no entrainment and 1 represents complete entrainment of gait cycle by tACS. To investigate whether gait cycle was entrained to a specific phase, the averaged MPDs of tACS were calculated and represented by complex numbers. The phase of the averaged MPDs were regarded as the MPDAvg.

For assessment of rhythmic sensation, analytic signals of tACS currents were acquired by the bandpass filtered currents following Hilbert transform in the same way as analysis of the entrainment effects described before. The instantaneous phase of the time to push the button was calculated during 5 min of recording. All instantaneous phases were represented by complex numbers and averaged. The absolute value was regarded as the PSI, respectively.

To investigate a relationship of PSIs and MPDs with changes in corticospinal excitability and walking parameters in tACS-Gait, correlation coefficients were calculated between the PSIs, the MPDs, the change ratios of the MEP amplitudes from the left TA muscles, the gait speed and the left leg strides.

### Statistical analysis

Complete-case analysis was performed with listwise deletion. The MEP amplitude, CSP duration, and the 10-m walk test parameters were subjected to two-way repeated-measures analysis of variance (ANOVA) with Condition [Time (pre, post0 and post1) × Condition (tACS-Gait, Sham-Gait, and tACS-Rest)] as a within-subject factor. Furthermore, they were subjected to two-way repeated-measures ANOVA [Time (pre, post0 and post1) × Order (session order: 1^st^, 2^nd^ and 3^rd^)] as a within-subject factor to investigate whether the session order had an effect on the results. Pre-stimulus EMG activity (RMS × time) in CSP measurement was subjected to one-way repeated-measures ANOVA with time (pre, post0, and post1) as a within-subject factor under each condition (tACS-Gait, Sham-Gait, and tACS-Rest).

Individual PSIs for evaluation of the entrainment effect and of the rhythmic sensation and the averaged PSI for the entrainment effects were assessed for statistical significance by nonparametric testing against an empirical null-distribution by using Matlab program (Matlab 2014b, The MathWorks, Inc., USA). The null-distribution was constructed by computing the statistic PSI over randomly oriented unit vectors, which were repeated 100,000 times according to the Bootstrap methods [33, 34]. To investigate whether MPDAvg was significantly close to a specific phase, the MPDAvg was subjected to the V-test of the circular statistics [35, 36]. Significance was accepted at the 5% confidence level (*p* < 0.05).

If necessary, the Greenhouse–Geisser correction was used to adjust for the sphericity, changing the degrees of freedom using the correction coefficient epsilon. The Bonferroni correction for multiple comparisons was used for the *post hoc* t-test. Effects were considered significant at *p* < 0.05. All data are expressed as the mean ± SD unless otherwise indicated. The JMP statistical package (JMP Pro 12.2, SAS Institute Inc., USA) was used for each of the 259 analyses unless otherwise described.

## Results

There were no adverse effects of TMS and tACS with a constant DC offset. No subjects reported phosphenes, vertigo, or skin irritation from stimulation.

For investigation of relationship between phase of stimulation current and gait cycle, the recording of accelerometer data was stopped in the middle of the experiment in the six subjects, because of the failure of accelerometer sensors. The data from the remaining 15 subjects were used for each statistical test by way of complete-case analysis.

As for the evaluation of the rhythmic sensation, the average number of the button push (± SD) was 6.78 ± 10.27 in the nine subjects. The remaining six subjects did not push the button since they did not feel any sensation during the stimulation. No participants exhibited significant PSI [average PSI (± SD): 0.38 ± 0.28, 95% confidence interval (CI): 0.292–0.997], suggesting that they did not sense the rhythmicity of stimulation during tACS.

During the interventions, the average (±SD) speed of treadmill walking for 10 min in the 15 subjects was 3.34 ± 0.39 km/h under the experimental conditions of tACS-Gait and Sham-Gait. The average frequency of tACS with a constant DC offset to match stride frequency was 0.94 ± 0.08 Hz for all three experimental conditions (tACS-Gait, Sham-Gait, and tACS-Rest).

### Effects of tACS with a constant DC offset on gait parameters

The average (± SD) of the 10m walking speed and of the left stride length were shown in Table 1.

**Table 1:** Gait parameters.

10 meter-walk test									
	tACS-Gait			Sham-Gait			tACS-Rest		
**walk speed (m/sec)**									
pre	1.28	±	0.20	1.31	±	0.21	1.31	±	0.20
post0	1.33	±	0.15	1.30	±	0.17	1.27	±	0.19
post1	1.39	±	0.16	1.32	±	0.20	1.32	±	0.22
**stride length (cm)**									
pre	131.1	±	13.6	133.3	±	15.6	133.1	±	12.5
post0	135.5	±	12.2	133.2	±	13.7	130.8	±	11.9
post1	137.7	±	11.4	133.5	±	15.6	133.1	±	14.4

The average (± SD) of the 10m walking speed and of the left stride length were shown.

**Fig 2:**
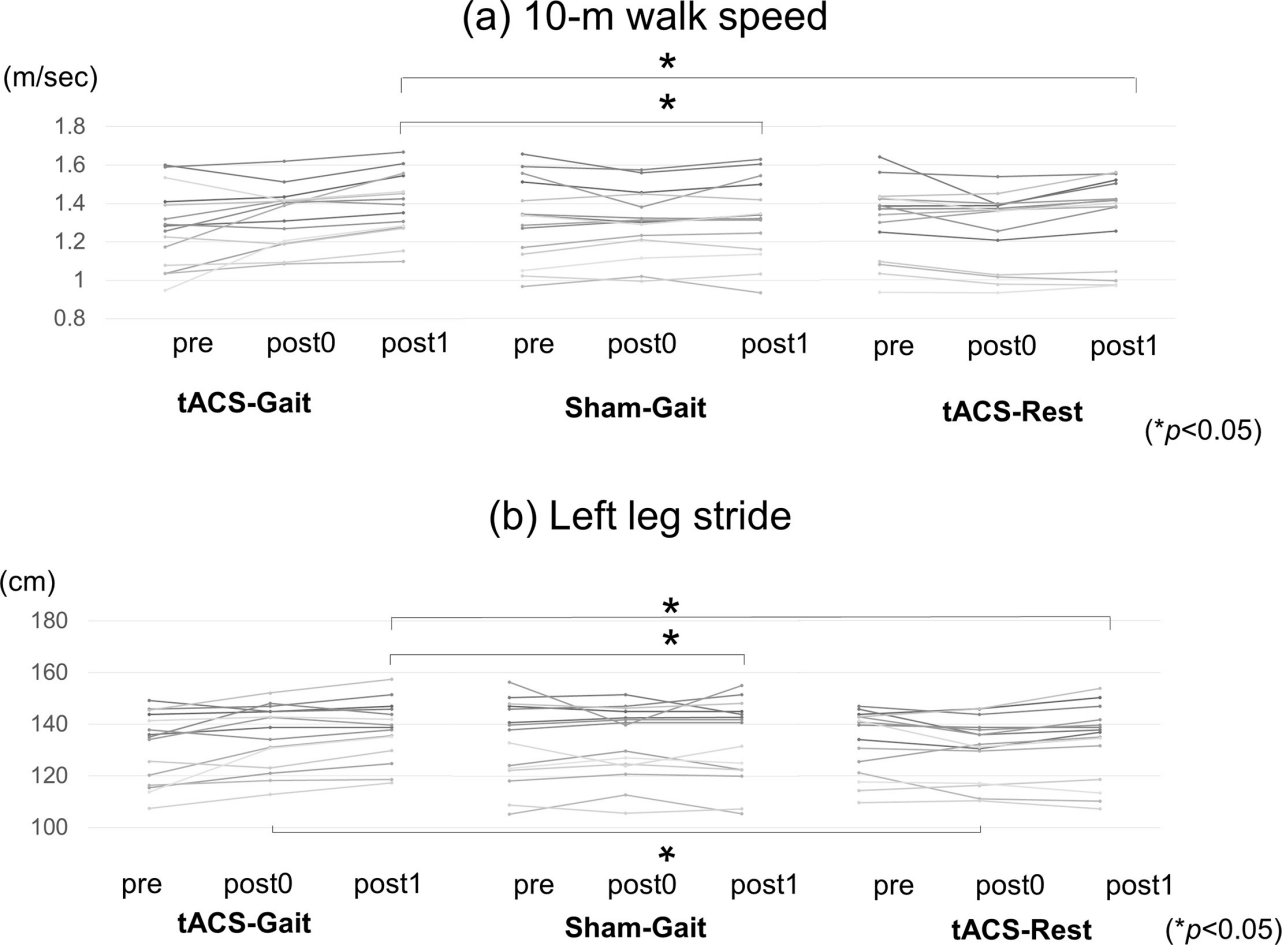
The 10-m walk speed and the left leg stride. The walk speed (a) and of the left leg stride (b) were shown in the tACS-Gait, Sham-Gait and tACS-Rest conditions.

There was a significant difference in the self-paced 10-meter walk speed and in the left leg stride length among tACS-Gait, Sham-Gait, and tACS-Rest conditions (10-m walk speed: F(4, 112) = 3.98, *p* = 0.005, left leg stride length: F(4, 112) = 3.79, *p* = 0.006 by repeated measures ANOVA). The 10-meter walk speed was significantly increased 30 min after the tACS -Gait condition compared with that after the Sham-Gait (*p* = 0.009, by *post hoc* t-test) and tACS -Rest conditions (*p* = 0.018). No differences were found between the tACS-Gait and Sham-Gait conditions at post0 (*p* = 0.444), between tACS-Gait and tACS-Rest conditions at post0 (*p* = 0.051) and between the Sham-Gait and tACS-Rest conditions at post0 and post1 (at post0, *p* = 0.604 and at post1, *p* = 0.809, respectively; Fig 2A). The left leg stride was significantly increased 30min after the tACS -Gait condition compared with that after the Sham-Gait (*p* = 0.025) and immediately (post0) and 30 min (post1) after the tACS -Gait condition compared with that after the tACS -Rest condition (at post0, *p* = 0.032 and at post1, *p* = 0.022). No differences were found between the tACS-Gait and Sham-Gait conditions at post0 (*p* = 0.597) and between the Sham-Gait and tACS-Rest conditions at post0 and post1 (at post0: *p* = 0.332 and at post1: *p* = 0.959, respectively; Fig 2B).

The order of the three session had no significant effect (10-meter walk speed: F(4, 112) = 0.662, *p* = 0.619, left leg stride length: F(4, 112) = 0.518, *p =* 0.722).

### Effects of tACS with a constant DC offset on corticospinal excitability within the right M1 foot area

The average (± SD) of the MEP amplitudes with SI1mV was shown in Table 2.

**Table 2:** MEP amplitudes with SI1mV from the bilateral TA and GC muscles.

MEP amplitudes with SI1mV (mV)									
	tACS-Gait			Sham-Gait			tACS-Rest		
**left TA**									
pre	0.70	±	0.17	0.82	±	0.30	0.76	±	0.27
post0	0.93	±	0.35	0.80	±	0.29	0.79	±	0.46
post1	1.14	±	0.39	0.89	±	0.45	0.72	±	0.36
**left GC**									
pre	0.62	±	0.23	0.54	±	0.21	0.55	±	0.25
post0	0.52	±	0.19	0.52	±	0.24	0.65	±	0.40
post1	0.63	±	0.35	0.58	±	0.27	0.60	±	0.39
**right TA**									
pre	0.83	±	0.31	0.82	±	0.30	0.81	±	0.31
post0	0.77	±	0.38	0.86	±	0.43	0.92	±	0.49
post1	0.97	±	0.51	0.91	±	0.38	1.03	±	0.63
**right GC**									
pre	0.56	±	0.26	0.61	±	0.31	0.58	±	0.17
post0	0.50	±	0.22	0.59	±	0.36	0.52	±	0.22
post1	0.56	±	0.27	0.68	±	0.29	0.61	±	0.30

The average (± SD) of the MEP amplitudes with SI1mV was shown.

**Fig 3:**
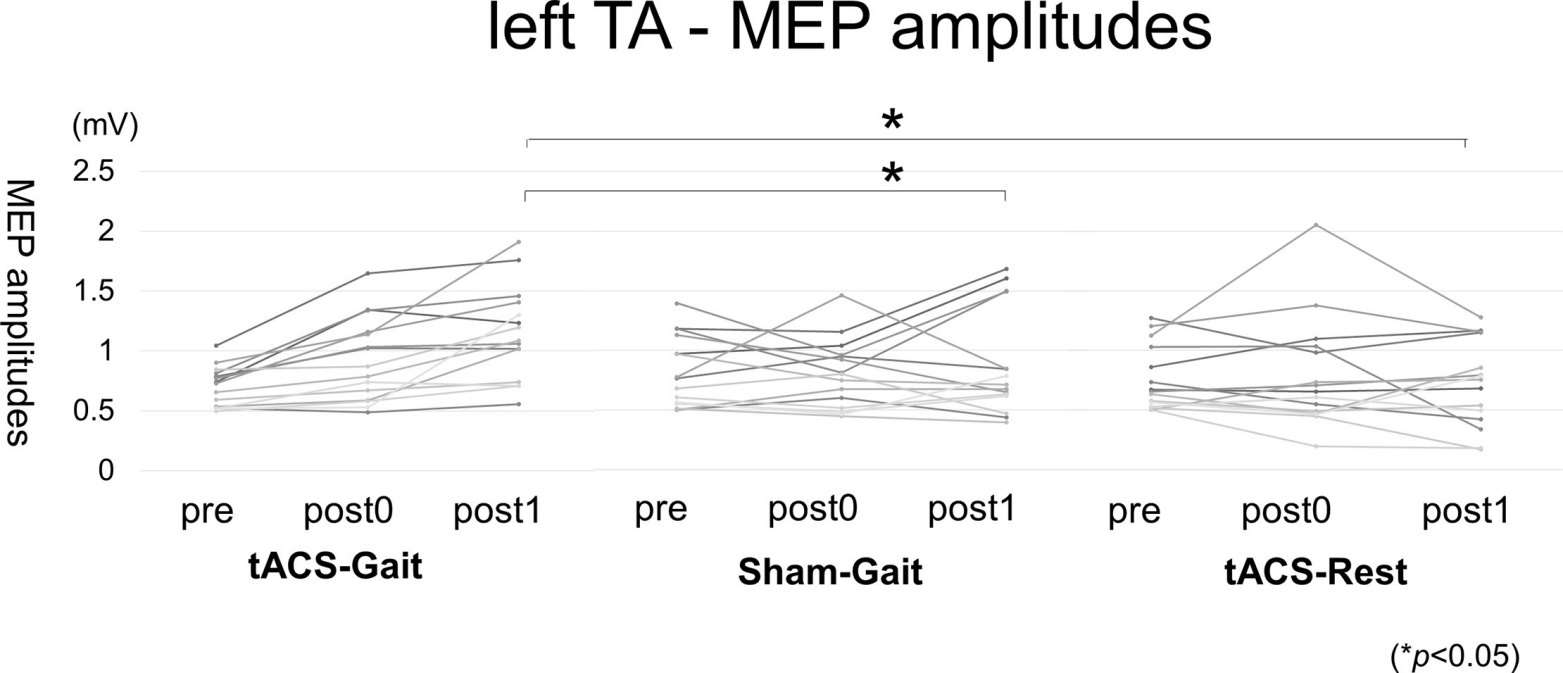
Changes of the left TA MEP amplitudes. The left TA MEP amplitudes were shown. The amplitude was significantly greater (indicative of enhanced corticospinal excitability) both immediately (post0) and 30 min (post1) after tACS-Gait compared with that after Sham-Gait and tACS-Rest.

There was a significant difference in the MEP amplitude of the left TA muscle among the three conditions (F(4, 112) = 4.75, *p* = 0.0014). Pair-wise comparisons revealed a significant increase in MEP amplitude of the left TA muscle 30 min after the tACS -Gait condition compared to the Sham-Gait condition (*p* = 0.007) and the tACS -Rest condition (*p* < 0.001; Fig 3), but no difference was found between the tACS-Gait and Sham-Gait conditions at post0 (*p* = 0.289), between tACS-Gait and tACS-Rest conditions at post0 (*p* = 0.220) and between the Sham-Gait and tACS -Rest conditions at post0 and post1 (at post0: *p* = 0.886 and at post1: 0.087, respectively). In contrast to the left TA muscle, there were no significant differences in the MEP amplitudes from the right TA and left and right GC muscles among the conditions (right TA muscle: F(4, 112) = 0.711, *p* = 0.586, left GC muscle: F(4, 112) = 1.43, *p* = 0.230, right GC muscle: F(4, 112) = 0.266, *p* = 0.900). The order of the three session had no significant effect (left TA muscle: F(4,112) = 1.40, *p* = 0.238, right TA muscle: F(4,112) = 0.677, *p* = 0.609, left GC muscle: F(4,112) = 0.575, *p* = 0.681, right GC muscle: F(4,112) = 1.80, *p* = 0.133).

For evaluating inhibitory networks in the M1, the CSP was measured. The integral values (μV·sec) of RMS in pre-stimulus EMG activity for 100 msec are shown in Table 3. There were no significant differences before and after each intervention (tACS-Gait: left TA muscle: F(2, 28) = 1.38, p = 0.269, right TA muscle: F(2, 28) = 1.72, p = 0.198, left GC muscle: F(2, 28) = 1.50, p = 0.241, right GC muscle: F(2, 28) = 0.325, p = 0.726, Sham-Gait: left TA muscle: F(2, 28) = 2.16, p = 0.134, right TA muscle: F(2, 28) = 1.54, p = 0.232, left GC muscle: F(2, 28) = 0.586, p = 0.563, right GC muscle: F(2, 28) = 2.52, p = 0.099, tACS-Rest: left TA muscle: F(2, 28) = 0.18, p = 0.836, right TA muscle: F(2, 28) = 3.26, p = 0.053, left GC muscle: F(2, 28) = 1.83, p = 0.180, right GC muscle: F(2, 28) = 0.179, p = 0.837).

**Table 3:** Pre-stimulus EMG activity.

	tACS-Gait			Sham-Gait		tACS-Rest			
**left TA**									
pre	9583	±	1366	11467	±	2067	10190	±	1213
post0	9642	±	1232	10308	±	2126	9886	±	959
post1	10198	±	1354	13121	±	2282	10288	±	1240
**right TA**									
pre	11467	±	2446	11330	±	2246	10521	±	1250
post0	10308	±	1223	10730	±	2262	11331	±	1216
post1	13121	±	2028	12734	±	2811	12490	±	1652
**left GC**									
pre	7491	±	1066	8213	±	1058	6903	±	611
post0	7055	±	831	8920	±	900	7879	±	1061
post1	7900	±	1027	8791	±	1080	7882	±	964
**right GC**									
pre	8220	±	980	8591	±	870	8927	±	1313
post0	8440	±	842	10538	±	1307	9342	±	1506
post1	8670	±	939	9106	±	1420	8867	±	859

(Average ± SEM) (μV⋅sec)

Pre-stimulus EMG activity was not significantly changed before and after the interventions in the CSP measurement.

The average (± SD) of the CSP was shown in Table 4.

**Table 4:** The CSP from the bilateral TA and GC muscles.

CSP (msec)									
	tACS-Gait			Sham-Gait			tACS-Rest		
**left TA**									
pre	196.7	±	23.8	202.8	±	21.0	188.2	±	19.3
post0	194.9	±	25.6	194.0	±	33.3	188.1	±	24.6
post1	210.5	±	29.5	204.1	±	25.9	192.1	±	22.2
**right TA**									
pre	198.2	±	24.1	206.4	±	26.7	198.0	±	32.4
post0	189.6	±	34.3	195.3	±	30.6	199.4	±	31.9
post1	198.9	±	30.3	213.1	±	36.6	196.3	±	36.4
**left GC**									
pre	173.6	±	18.6	175.7	±	20.7	170.4	±	24.8
post0	173.7	±	22.6	174.7	±	24.0	174.7	±	20.2
post1	180.5	±	22.0	178.5	±	26.7	166.2	±	22.8
**right GC**									
pre	176.8	±	31.0	175.1	±	34.0	177.3	±	33.0
post0	177.1	±	29.9	172.4	±	29.3	175.0	±	26.9
post1	180.1	±	29.4	182.3	±	32.6	178.3	±	28.1

The average (± SD) of the CSP was shown.

No significant differences were found in the CSP of the left and right TA and the left and right GC muscles among conditions (left TA: F(4,112) = 0.732, *p* = 0.572; right TA: F(4,112) = 1.31, *p* = 0.270; left GC: F(4,112) = 1.12, *p* = 0.349; right GC: F(4,112) = 0.239, *p* = 0.916). The order of the three session had no significant effect (left TA muscle: F(4,112) = 0.461, *p* = 0.765, right TA muscle: F(4,112) = 0.383, *p* = 0.820, left GC muscle: F(4,112) = 0.930, *p* = 0.449, right GC muscle: F(4, 112) = 0.677, *p* = 0.609).

### Relationship of corticospinal excitability and walking function

**Fig 4:**
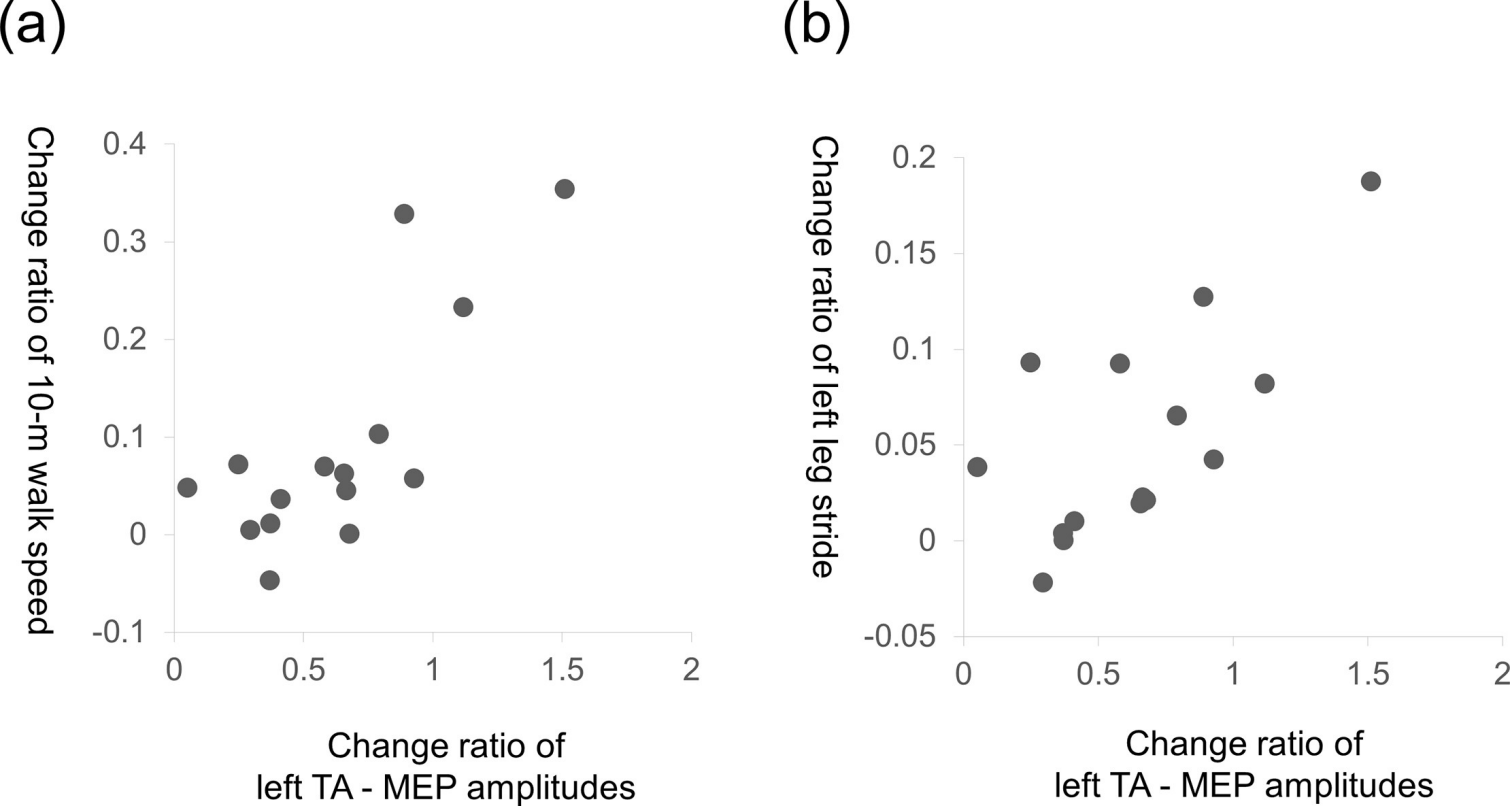
Correlation between change ratio of the left TA MEP amplitudes, walk speed and left leg stride. (a) The change ratios (post1/pre) of the MEP amplitudes from the left TA muscles were positively correlated with those of the 10-m walk speed. (b) The change ratios (post1/pre) of the MEP amplitudes from the left TA muscles were positively correlated with those of the left leg stride.

The change ratios (post1/pre) of the MEP amplitudes from the left TA muscles were positively correlated with those of the gait speed (*r* = 0.767, *p* = 0.001) and those of the left leg strides (*r* = 0.678, *p* = 0.005) (Fig 4A and 4B), while the change ratios (post0/pre) of those parameters were not correlated (ratios of MEP amplitudes and gait speed: *r* = − 0.213, *p* = 0.446, ratios of MEP amplitudes and gait speed: *r* = − 0.066, *p* = 0.816).

### Relationship between phase of stimulation current and gait cycle in the tACS-Gait condition

The mean number of gait cycles counted on the left leg was 530.5 ± 63.2, and the mean frequency of tACS was 0.95 ± 0.08 Hz over 10 min. The 95%CI for the statistically significant PSI was approximately 0.08 (0.0706–0.0841). Twelve subjects exhibited significant PSI, whereas three did not (Table 5). The average PSI of all the subjects was 0.15 ± 0.13, representing significant entrainment compared with the null distribution (0.05 confidence level = 0.441, *p* < 0.05). To investigate whether gait cycle was entrained to a specific phase, the averaged MPDs of tACS were calculated with complex numbers (the calculated phase represented by MPDAvg). The MPDAvg was −86.1°. According to the V-test, MPDAvg was significantly approximate to − 90° (*p* = 0.045 < 0.05).

**Table 5:** Individual PSI and MPD.

Subject #	Number of the gait cycle	tACS Freqency (Hz)	PSI	*p<0.05	MPD
					(-180<θ≦180°)
1	445	0.92	0.061		(7.1)
2	530	0.88	0.060		(109)
3	457	0.77	0.196	*	-87.9
4	598	0.94	0.341	*	-99.8
5	423	0.97	0.097	*	-55.2
6	560	0.93	0.202	*	-75
7	598	0.94	0.122	*	169.2
8	573	0.9	0.241	*	-96.2
9	562	1.07	0.010		(159.1)
10	586	1.03	0.072	*	-105.5
11	581	1	0.080	*	-101.1
12	476	0.84	0.509	*	-29
13	535	1.06	0.083	*	-94.6
14	587	0.99	0.092	*	-93
15	447	0.95	0.083	*	-108.4

The individual gait cycles over 10 min, frequency of tACS, PSI and MPD are shown. In the six subjects, the recording of accelerometer data was stopped in the middle of the experiment. Twelve subjects exhibited significant PSI, whereas three did not.

* means p < 0.05.

### Relationship of PSI and MPD with corticospinal excitability and walking function

Neither of the PSIs nor the MPDs were correlated with the change ratios (post1/pre) of the MEP amplitudes of the left TA muscle (PSI: *r* = − 0.142, *p* = 0.613 and MPD: *r* = 0.050, *p* = 0.860), 10-m walk speed (PSI: *r* = − 0.243, *p* = 0.383 and MPD: *r* = 0.001, *p* = 0.997) and left leg stride (PSI: *r* = − 0.400, *p* = 0.139 and MPD: *r* = − 0.128, *p* = 0.650).

## Discussion

Anodal transcranial patterned stimulation to the right M1 foot area increased the walk-speed and the stride length, and enhanced corticospinal excitability of the left leg TA (foot flexor) muscle when combined with walking. Moreover, gait-combined anodal transcranial patterned stimulation but not sham stimulation or anodal transcranial patterned stimulation at rest enhanced left TA corticospinal excitability for at least 30 min after intervention. The degree of enhancement of the left TA corticospinal excitability was positively correlated with that of the enhancement of gait parameters. In 12 of 15 participants tested, gait-combined anodal transcranial patterned stimulation to the right M1 foot area also entrained the gait cycle. These findings suggest that anodal patterned brain stimulation enhanced corticospinal input to the left leg muscles and increased gait speed and left leg stride as well as entrained neuronal activity within the right M1 foot area. This is the first demonstration that anodal patterned noninvasive cortical stimulation can induce a prolonged change in gait function, presumably by possibly activating synaptoplastic mechanisms in corticospinal pathways originating in the M1 foot area [37–41].

During human bipedal gait, corticospinal activity is higher in leg flexors such as the TA muscle than in extensors such as the GC muscle [4, 42]. Our findings suggest that anodal transcranial patterned stimulation enhances corticospinal input to the flexor muscles involved in the swing phase by inducing long-term potentiation (LTP)-like effects on membrane potentials and synaptic connections [37–41, 43–45] and that corticospinal excitability is increased specifically for leg flexors such as the TA muscles. Patients with stroke lesions involving the sensorimotor cortex or the internal capsule often show walking deficits like foot-drop due to decreased activation of the ankle flexors and increased tonus in the ankle extensors [7]. One therapeutic aim of treatment for this deficit is to improve paretic flexor function. Most previous approaches targeted peripheral nerves and muscles to indirectly influence corticospinal tracts [46, 47]. Our findings suggest a possible alternative approach to directly improve output from the injured corticospinal tract by potentiation.

In our findings, activity-dependent effects were likely induced by the tACS-Gait intervention because neither Sham-Gait nor tACS-Rest showed any observable changes. Previous reports showed that online stimulation, that is concurrent brain stimulation with trainings could enhance between-session consolidation of learning compared with offline stimulation [48, 49], indicating that associative LTP-like effects were induced through the Hebbian rule in training-specific neuronal circuits as activity-dependent effects [50, 51]. We found that the degree of increase in the left TA corticospinal excitability was positively correlated with that of the enhancement of gait parameters, suggesting that increased corticospinal excitability might have contributed to alteration of walking ability. The tACS-Gait intervention might be an effective approach as a training combined with brain stimulation for functional recovery of patients with brain damages [52–54].

We found the time-dependent aftereffects of the tACS-Gait intervention. Significant changes in corticospinal excitability and in walk speed were found 30 min after, not immediately after the intervention. Previous studies reported time-dependent consolidation of anodal tDCS on the M1 area [55–58]. The tDCS retained the acquired visuomotor skill more than 3 hours after the training, while the sham stimulation groups showed loss of the skill, and there was no significant difference between the tDCS and sham stimulation groups during and 15 min after the training [57]. A consolidation-like mechanism might have worked in our findings, although 30 minutes appeared a rather short time to induce consolidation. Further investigation of time-dependency of the effects is necessary in future. If the performance gains are retained by the stimulation, it is useful in application to clinical settings in rehabilitation since it can lead to improve gait function in daily life activity, not only during the training.

We did not observe any changes in the CSP duration after tACS-Gait, Sham-Gait and tACS-Rest. Previous studies showed that high-frequency rTMS on the affected M1 area induced prolongation of CSP duration [59, 60] and improvement of gait parameters [61] in patients with Parkinson’s disease who often show impaired M1 inhibitory system [62]. It suggested that GABA_B_ receptor-mediated intracortical inhibitory neurons might have not been involved in gait-related cortical activity and unmodulated by tACS with positive DC offset in healthy subjects.

In our study, an entrainment effect was observed between the patterned brain stimulation and the individual gait cycle in 12 of 15 subjects. Previous studies showed that the patterned brain stimulation such as tACS with or without DC offset could entrain the brain oscillation when it applied at a frequency similar with that of endogenous specific band frequency [16–22]. During human gait, there is significant coherence between the M1 leg area and TA muscles during the swing phase of walking [6] and alpha- and beta-band power in the sensorimotor cortex is significantly increased prior to the toe-off [24]. Previous studies have found that low frequency brain oscillations can be modulated with higher frequency oscillation through cross-frequency coupling actions such as phase–amplitude coupling and phase–phase coupling [63, 64] and that slow (0.75 Hz) frequency tACS increases and expands theta oscillations (4–8 Hz) into widespread brain areas [14]. Those gait cycle-dependent M1 oscillations may have been entrained through cross-frequency coupling and tACS-Gait intervention, leading to modulation of individual gait rhythms. In future, EEG recordings during gait would be necessary to reveal a linkage between brain oscillations during gait and entrainment of gait rhythms by tACS-Gait intervention.

While oscillatory brain activity was entrained by patterned brain stimulation, the aftereffects seem independent of phase-continuity and neither due to prolonged phase alignment nor frequency synchronization to the exact stimulation frequency [65–68]. In the present study, there was no correlation of the degree of phase synchronization and differences with the changes of corticospinal excitability and those of gait parameters.

Our findings suggested that the aftereffects were induced by combination of anodal stimulation with a constant positive DC offset and gait exercises through a frequency-independent plasticity. We could not elucidate an effect of the stimulation pattern by the current procedures and results.

In our study, the phase difference between the stimulation and initiation of the stance phase on the target side was significantly close to −90°. It is likely that this specific phase difference was stabilized by the brain-tDCS interaction. One recent study found that tDCS similar in frequency to Parkinsonian tremor could exacerbate the tremor at one phase difference and suppress it at another [23]. When the patterned tDCS was synchronized with the tremor cycle at the suppressive phase difference, it markedly attenuated tremor by modulating oscillatory cortical activities at tremor frequency [23, 69]. The phase difference that we found may be optimal for entraining the gait-related cortical rhythm since the increasing electrical current with sinusoidal form appears to match the increasing swing phase-related cortical activity [4–6]. In the future, patterned tDCS synchronized with the gait cycle of the paretic leg at a specific phase difference could be used to enhance gait-related phasic cortical activity, leading to improvement of gait function in patients with central nervous system (CNS) disorders. Gait-combined brain stimulation could be a promising therapy for gait disturbances caused by brain damage.

It remains unclear what specific parameters of the patterned tDCS are most critical for the modulation of rhythmic brain activity. For instance, it is unknown whether patterned tDCS with a constant DC offset is more effective for driving intrinsic rhythmicity than patterned tDCS without offset. The net current polarity, which is the offset of the patterned tDCS, determines the direction of shift in cortical excitability, similar to the polarity-dependent effects of conventional tDCS using a fixed current [13]. Therefore, it may be better to use tACS with a constant positive DC offset to enhance neuronal activity in the lesioned brain. We suggest that our method could be applied for the neurorehabilitation of patients with gait disorders caused by CNS lesions.

There are several limitations in the present study. Firstly, since experimenters’ expectation might influence the subjects’ walking speed [70], the study with double blind design would be needed in the future. The Sham-Rest condition with a factorial design would be preferable for the next step, which was found to be stable in a few subjects in a preliminary experiment, as expected. The relationships between MEP variability rates for the TA muscles and the rates of post-stimulus walking speed and stride variability were verified although the relationship appeared to be weak due to the large variance. Further investigation would be necessary about the effects on spinal excitability by concurrently measuring MEPs, EMGs, F waves and H reflexes during gait [71–75], to confirm that the changes in MEPs in the current study is not due to changes in spinal excitability.

When walking speed and stride length increase, the timing of toe-off becomes faster, along with larger EMG activities of the TA muscles during the swing phase [76–80]. In the present study, enhanced TA muscles might have made the timing of foot clearance faster, resulting in a faster start to the swing phase. Kinematic data including timing of foot clearance, flexion angles of limb-joints, and leg muscle activities during both stance and swing phases would be useful in revealing the effects of the tACS-Gait intervention in more detail [81–83].

Our findings suggested that the gait parameters were not changed from the effect of the patterned stimulation, but rather from the anodal stimulation with a positive DC offset combined with the gait exercise. To investigate detailed effects of patterned stimulation on gait-related activities, comparison of the tACS and the tDCS combined with gait would be necessary in future.

This is the first demonstration that patterned brain stimulation applied over the M1 foot area during gait can enhance the excitability of corticospinal tracts controlling foot flexor muscles, thereby altering gait parameters (notably walk speed and stride length). Furthermore, it entrained individual gait cycle in some subjects. We speculate that this patterned stimulation could be a potentially promising approach for treatment of patients with gait disturbance caused by CNS disorders.

## Abbreviations

ANOVA: analysis of variance
BDNF: brain-derived neurotrophic factor
CI: confidence interval
CNS: central nervous system
CSP: cortical silent period
DC: direct current
GC: gastrocnemius
LTP: long term potentiation
M1: primary motor cortex
MEP: motor evoked potential
MPD: mean phase difference
PSI: phase synchronization index
RMS: root mean square
rMT: resting motor threshold
TA: tibialis anterior
tACS: transcranial alternating current stimulation
tDCS: transcranial direct current stimulation
